# Supplementary material for: Characterization of Glyceollins as Novel Aryl Hydrocarbon Receptor Ligands and Their Role in Cell Migration
Source: Int J Mol Sci. 2020 Feb 18;21(4):1368. doi: 10.3390/ijms21041368 (PMC7072876; doi:10.3390/ijms21041368)
Supplement: Supplementary file 1 [file ijms-21-01368-s001.pdf]

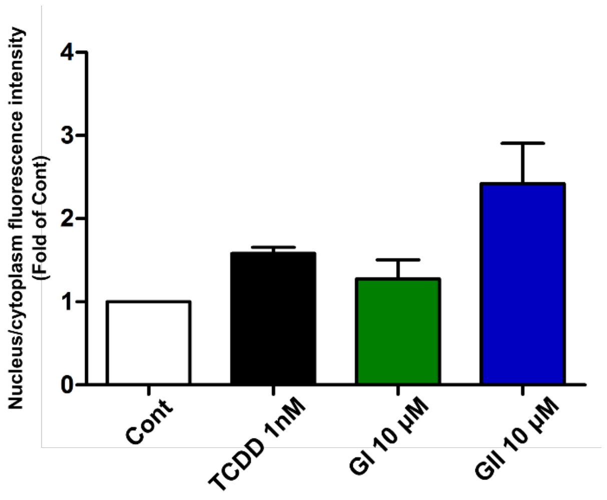

Supplementary figure 1. Nucleus/cytoplasm fluorescence intensity of AhR in HepG2 treated with DMSO at 0.1% (*v/v*) as Cont, TCDD 1nM, GI 10 µM or GII 10 µM for 24 h. Fluorescence intensity in nucleus and cytoplasm of in total 3074 cells distributed in the four treatments was measured using the software ImageJ from two independent experiments. For each experiment, nucleus/cytoplasm intensity of treated conditions is presented in fold change compared with nucleus/cytoplasm intensity of Cont condition. Data is represented as the mean ± SD.

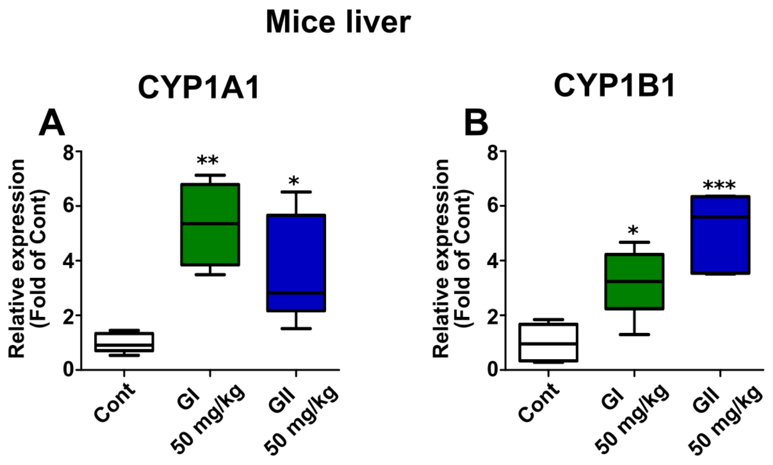

Supplementary figure 2. Glyceollin treatment increases the level of CYPA1 and CYP1B1 in vivo in mice liver. Mice liver came from ovariectomized RjOrl SWISS female treated by subcutaneous injection for 3 consecutive days with glyceollin I or II 50 mg/kg/day. The relative expression of CYP1A1 (**A**) and CYP1B1 (**B**) was assessed by real-time PCR and normalized to the expression of the housekeeping gene GAPDH. The results are expressed as the change fold of each group compared to the control group and are represented as the box and whiskers (10-90 percentile) of at least 4 animals per group. Statistical analyses were performed with one-way ANOVA followed by Dunnett's post hoc test. * p-value < 0.05, ** p-value < 0.01, *** p-value < 0.001 are considered to be significantly different with the control group.

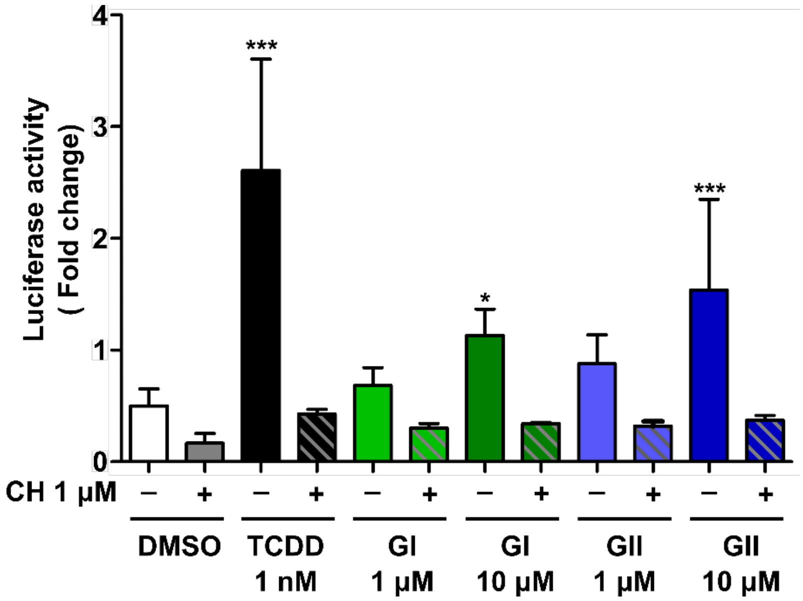

Supplementary figure 3. Glyceollins activate aryl hydrocarbon receptor (AhR) bound to XRE-containing CYP1A1. AhR-mediated transcriptional activity was analyzed by transfecting cells with an XRE-CYP1A1 promoter pHu-1A1-FL luciferase reporter plasmid and a CMV-β-galactosidase plasmid as a control for transfection efficiency. MCF-7 cells were treated for 24 h with DMSO at 0.1% (*v*/*v*) (white) to serve as the control (Cont), TCDD (black) at 1 nM or two different concentrations of GI (green) and GII (blue), alone or in combination with 1 µM CH223191 (CH), an AhR antagonist. The results are expressed as the fold change in luciferase activity compared with that in the control and are represented as the mean ± SD. The experiment was done 3 times in triplicate. Statistical analyses were performed with one-way ANOVA followed by Dunnett's post hoc test. * p-value < 0.05, *** p-value < 0.001 are considered to be significantly different with the control group.

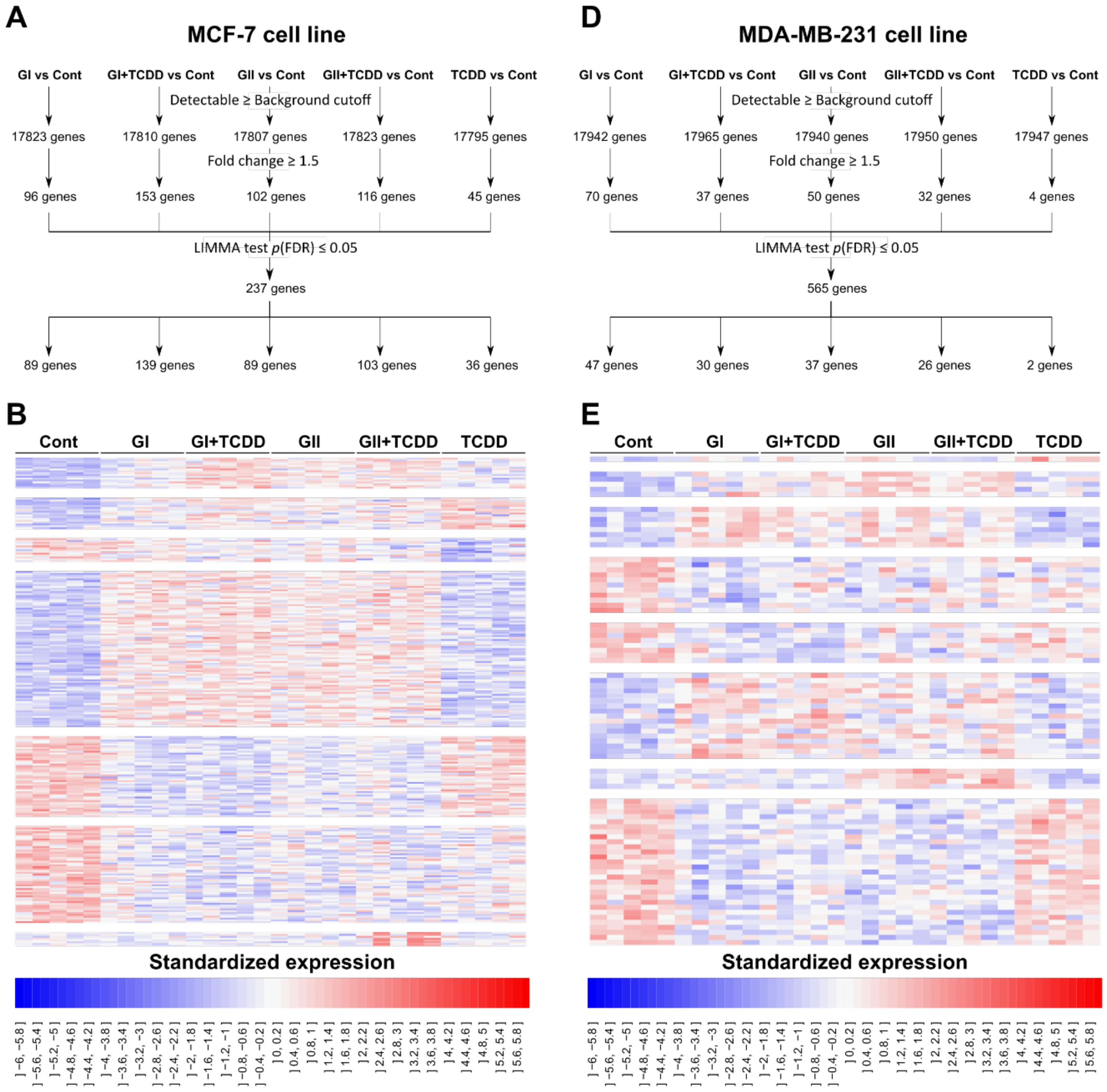

C

Biological process	Number of genes	p(BH)
Small molecule biosynthetic process	32	1.81E-09
Small molecule metabolic process	56	1.04E-08
Oxidation-reduction process	33	5.97E-06
Regulation of biological quality	77	0.0001
Exocytosis	25	0.0007
Secretion by cell	26	0.0055

F

Biological process	Number of genes	p(BH)
Cellular response to organic substance	24	0.0139
mRNA processing	11	0.0139
mRNA metabolic process	13	0.0139
RNA splicing	10	0.0149
Regulation of mRNA processing	6	0.0199
RNA metabolic process	22	0.0400

Supplementary figure 4. Transcriptomic analysis, selection and clustering of differentially expressed genes in the MCF-7 cells and MDA-MB-231 cells. MCF-7 **(A, B, C)** or MDA-MB-231 **(D, E, F)** cells were treated with 0.1 % (*v*/*v*) DMSO to serve as the control (Cont), 2,3,7,8-tetrachlorodibenzo-p-dioxin (TCDD) at 1 nM, glyceollin I (GI) or glyceollin (GII) at 10 μM alone or in combination with TCDD for 24 h. Total RNA was extracted, labeled, reverse transcribed and sequenced. **(A, D)** The differentially expressed genes for each treatment were selected in comparison to the control levels. Then all probes with an intensity signal greater than the overall median and a fold change ≥ 1.5 were selected. The genes were then grouped together and subjected to a LIMMA test; and only those probes with a *p*-value ≤ 0.05 were retained, resulting in a total of 237 differentially expressed genes for MCF-7 cells **(A)** and 83 differentially expressed genes for MDA-MB-231 cells **(D)**. **(B, E)** 237 differentially

expressed genes for MCF-7 cells (**B**) and 83 differentially expressed genes for MDA-MB-231 cells (**E**) were clustered on the basis of their expression patterns. **(C**, **F)** Main biological processes that the differentially expressed genes by all treated conditions compares with control involve in.

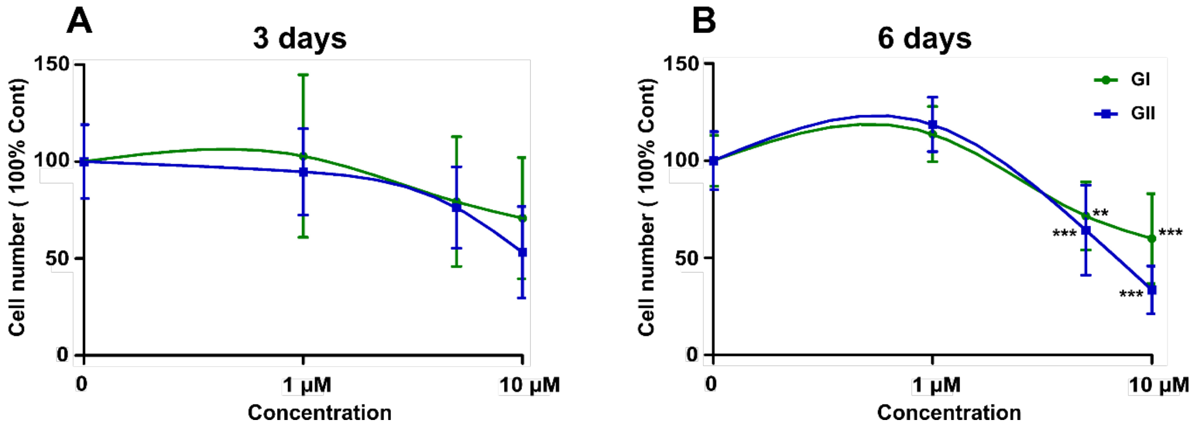

Supplementary figure 5. Effect of glyceollin I and II on the proliferation of the MDA-MB-231 cell line. MDA-MB-231 cells were treated with various concentrations of glyceollin I (GI) and glyceollin II (GII). Cell numbers were determined by counting after 3 days (**A**) and 6 days (**B**) of treatment. The experiment was done 3 times in triplicate. Statistical analyses were performed with one-way ANOVA followed by Dunnett's post hoc test. ** *p*-value < 0.01, *** *p*-value < 0.001 are considered to be significantly different with the control group.
